# Joint Toxicity of Different Heavy Metal Mixtures after a Short-Term Oral Repeated-Administration in Rats

**DOI:** 10.3390/ijerph14101164

**Published:** 2017-10-01

**Authors:** Hong Su, Zhou Li, Samuel Selorm Fiati Kenston, Hongbo Shi, Yafei Wang, Xin Song, Yuanliang Gu, Tabatha Barber, Joni Aldinger, Baobo Zou, Min Ding, Jinshun Zhao, Xialu Lin

**Affiliations:** 1 Department of Preventative Medicine, Zhejiang Key Laboratory of Pathophysiology, Medicine School of Ningbo University, 818 Fenghua Road, Ningbo 315211, Zhejiang, China; suhong0908@126.com (H.S.); lizhou0601@163.com (Z.L.); newsamsel@yahoo.com (S.S.F.K.); wangyafei926@163.com (Y.W.); songxin3c@163.com (X.S.); gurice@yeah.net (Y.G.); zoubaobo@nbu.edu.cn (B.Z.);; 2 Ningbo Municipal Center for Disease Control and Prevention, Ningbo 315010, Zhejiang, China; shihb@nbcdc.org.cn; 3 Toxicology and Molecular Biology Branch, Health Effects Laboratory Division, National Institute for Occupational Safety and Health, Morgantown, WV 26505, USA; yga3@cdc.gov (T.B.); LIZ0@cdc.gov (J.A.); mid5@cdc.gov (M.D.); 4 School of Public Health, Key Laboratory of Environment and Gene Related Diseases of Ministry of Education, Health Science Center, Xi’an Jiaotong University, Xi’an 710049, Shaanxi, China

**Keywords:** heavy metals, heavy metal mixtures (HMMs), fish consumption, joint toxicity, SD rat

## Abstract

The systemic toxicity of different combinations of heavy metal mixtures (HMMs) was studied according to equivalent proportions of the eight most common detectable heavy metals found in fish consumption in the Ningbo area of China. The ion mass proportions of Zn, Cu, Mn, Cr, Ni, Cd, Pb, and Hg were 1070.0, 312.6, 173.1, 82.6, 30.0, 13.3, 6.6, and 1.0, respectively. In this study, 10 experimental groups were set as follows: M8 (Pb + Cd + Hg + Ni + Cu + Zn + Mn + Cr); M5 (Pb + Cd + Hg + Ni + Cr); M4A (Pb + Cd + Hg + Ni); M4B (Cu + Zn + Mn + Cr); M3 (Cu + Zn + Mn); Cr; Cu; Zn; Mn; and control. Sprague Dawley (SD) rats were orally treated with a single dose of each group every three days (10 times in total) for 34 days. After Morris water maze test, blood and tissue samples were collected to obtain biochemical, histopathological and western blot analysis. Results show abnormalities could be observed in different treatment groups, the M4B combination had the most significant change compared to all other groups. In conclusion, combination HMMs may have adverse effects on the hematologic, hepatic, renal and neurobehavioral function, and may also disturb electrolyte and lipid balance. Why M4B combination generated much higher toxic effects than any other combination mixtures or individual heavy metal needs to be further evaluated.

## 1. Introduction

According to the priority chemicals list [1], lead, mercury, cadmium, hexavalent chromium, and nickel are all toxic metals and have a high frequency of occurrence in the environment. Cadmium, chromium, lead, and mercury have high toxicities and rank among the priority metals that are of great public health significance [2]. Lead is toxic even at very low levels. Exposure to low levels of lead has been associated with behavioral abnormalities, learning impairment, decreased hearing, and impaired cognitive functions in humans and in experimental animals [3]. Cadmium and its compounds can induce disturbances in calcium metabolism, renal tubular dysfunction, or osteoporosis [4]. Epidemiological studies have shown that there exists a correlation between cadmium exposure and certain cancers [5]. Chromium is a widespread industrial compound. The soluble hexavalent chromium Cr (VI) as an environmental contaminant, is widely recognized as a carcinogen, mutagen, and teratogen toward humans and animals [6]. Nickel compound exposure can lead to nephrotoxicity, skin irritation and hypersensitivity [7]. Zinc, manganese and copper as common nutritional metallic elements, are beneficial to the body at moderate levels but may produce toxicity at high levels. Excess zinc exposure may induce toxic effects on the hematopoietic system, biochemistry and endocrine system function [8]. Manganese may lead to serious neurological symptoms, psychiatric, motor disturbances, and even result in a permanent neurological disorder [9]. Copper is a key constituent of the respiratory enzyme complex cytochrome C oxidase (COX) and is important in facilitating iron uptake. Excess copper exposure can lead to gastrointestinal symptoms and hepatocellular toxicity. Metals are systemic toxicants that are known to induce multiple organ damage, even at lower levels of exposure [10].

Experimental and epidemiological evidence indicates that combination effects generated by multi-heavy metals might be quite different from that induced by the same individual metal, because heavy metals at individual low acting concentrations can elicit higher toxicity on interactions with other environmental toxicants [11,12,13,14]. Goyer [15] has shown toxic metals have significant interactions with essential metals (iron, manganese, calcium) which can influence the essential metal status in the human body. Pandya *et al*. [14] showed that under similar dosages, when Pb and Cd are present together, the toxic effect is antagonized by co-exposure due to possible competition among Pb and Cd for hepatic accumulation. Damian *et al*. [16] suggested that when Clarias gariepinus was exposed to the metal mixture of zinc and copper, antagonism occurs at the ratio of 1:1 and synergism at the ratio of 1:2. Prato et al. [17] identified combined toxicity of dissolved copper-cadmium and mercury using the embryotoxicity test with Mytilus galloprovincialis, they concluded additive toxicity index (S) showed an antagonistic response. Xu *et al.* [18] tested additive effects of Pb + Cd + Cu + Zn mixture on sea urchin embryos and the toxicity was mainly determined by the combined action of bio-concentrations of metals.

Heavy metals are ubiquitous and generally persist in the environment. Biological accumulation in the food chain enables multi-heavy metal contaminants to magnify [19]. The fact is that human beings are normally exposed to multiple heavy metals simultaneously, through air, water or food but not individually. There are many researches on the binary heavy metal mixtures, but studies on the mixtures composed of three or more heavy metals are still insufficient. Experimental assessment on the joint toxicity of multi-heavy metal mixtures on particular organisms would be helpful for us to propose policies to protect the ecosystem and human health. Based on this fact, we evaluated the joint toxicities of different heavy metal combination mixtures through a short-term repeated oral administration in rats according to the corresponding proportion of the eight most common detectable heavy metals in fish consumed in the Ningbo area [20].

## 2. Materials and Methods

### 2.1. Materials 

Heavy metal compounds, lead acetate ((CH_3_COO)_2_Pb·3H_2_O, AR ≥ 99.5%), cadmium dichloride (CdCl_2_·2.5H_2_O, AR ≥ 99.0%), nickel dichloride (NiCl_2_·6H_2_O, AR ≥ 98.0%), manganese dichloride (MnCl_2_·4H_2_O, AR ≥ 99.0%), zinc sulfate heptahydrate (ZnSO_4_·7H_2_O, AR ≥ 99.5%), cupric sulfate (CuSO_4_·5H_2_O, AR ≥ 99.0%), and potassium dichromate (K_2_Cr_2_O_7_, AR ≥ 99.8%) were purchased from Sinopharm Chemical Reagent Co., Ltd (Shanghai, China). Methyl mercury chloride was purchased from Ehrenstorfer GmbH (affiliation, Germany). The biochemical and hematological analysis were performed by Hitachi 7600-110 autoanalyzer (Hitachi Ltd, Tokyo, Japan) and blood analyzer (Sysmex XT-1800i; Sysmex Co, Kobe, Japan), respectively. Water maze testing was performed using a MWM video analysis system (RD1101-MWM-G, Shanghai Mobiledatum Information Technology Co., Ltd., China). The monoclonal antibodies including p38, P-p38, JNK, p-JNK (phosphorylated JNK), NFκB, Nrf2, Akt, and GAPDH were obtained from Cell Signaling Technology (Danfoss, MA, USA). Sprague Dawley (SD) rats were obtained from Zhejiang Provincial Laboratory Animal Science Center (Hangzhou, Zhejiang, China).

### 2.2. Methods

#### 2.2.1. Experimental Design and Preparation

The proportion of each heavy metal used in this study for the HMMs is based on the estimation of the eight most common detectable heavy metals in fish consumed in the Ningbo area [20,21]. Among the eight heavy metals in the fish consumption, the mass percentage of Zn, Cu, Mn, Cr, Ni, Cd, Pb and Hg are 64.68%, 19.08%, 10.6%, 2.53%, 1.84%, 0.82%, 0.4% and 0.06%. The mass proportions of each heavy metal ions used are 1070.0 Zn, 312.6 Cu, 173.1 Mn, 82.6 Cr, 30.0 Ni, 13.3 Cd, 6.6 Pb, and 1.0 Hg, respectively [20,21]. The compounds used to represent the heavy metals include zinc sulfate heptahydrate, cupric sulfate, manganese dichloride, potassium dichromate, nickel dichloride, cadmium dichloride, lead acetate, and methyl mercury chloride metal salts. To prevent precipitation, each heavy metal compound was separately dissolved before use, and then diluted to the desired concentration by deionized water. The dosage for each group is shown in Table 1. Rats in the control group were given the same volume of deionized water as other groups. The M8 mixture contains eight heavy metals including Pb, Cd, Hg, Cu, Zn, Mn, Cr and Ni prepared according to their molecular proportion found in fish consumption in the Ningbo area. Based on previous research [21,22], the total exposure dose in the M8 group was set at 500 mg /kg body weight. The rats were orally given one time daily dose (50 mg/kg body weight) every three days, and a total of 10 times in 34 days. The exposure level of all the other individual metals or mixture groups were set up based on the M8 group including the dosage frequencies. Based on non-essential metals (Pb, Hg, Cd, Ni and Cr) or essential metals (Cu, Zn and Mn) to the human body [23,24], additional mixtures were prepared as: M3 (Cu + Zn + Mn), M4A (Pb + Hg + Cd + Ni), M4B (Cu + Zn + Mn + Cr), and M5 (Pb + Hg + Cd + Ni + Cr). We set Cu, Zn, and Mn as a separate group because they were found in the highest concentrations and their toxicities at such high doses were unknown. Cr was investigated separately because its toxicity has been well established at the concentration observed in this study. 

Healthy male SD rats (seven weeks old) were randomly divided into 10 groups (six rats in each group). The exposure and observation period was about 34 days. All SD rats were kept in stress free, clean, and animal-friendly conditions in a standard environment (relative humidity 60 ± 10%, room temperature 20 ± 2 °C, and 12 h light/dark cycle). Food and water were available ad libitum. Animal study protocols were approved by the Ningbo University Institutional Animal Care and Use Committee (AEWC-2014-102).

#### 2.2.2. Morris Water Maze Test

Animals were weighed every three days in order to follow their growth condition. Morris water maze (MWM) test was carried out four days before the end of the experiment. On the first three days, the hidden platform training was done where each rat was allowed 60 seconds in the water twice daily. The fourth day was for the hidden platform test and exploration with a time limit of 70 s. The MWM was a round pool (180 cm diameter, 60 cm depth) with black tank walls, filled with water mixed with black ink and maintained at a temperature of 22–24 °C. Visual cues were arrayed around four directions of the pool. The swim time, traveled distance and swim path around the pool was recorded during all the trials.

#### 2.2.3. Organ/Body Weight Coefficients, and Hematological, Biochemical, Electrolyte and Thyroxine Parameter Analysis 

At the end of the experiment each rat was sacrificed under anesthesia in slight diethyl ether. Blood was collected for hematological and biochemical analysis in anticoagulant or coagulation promoting tubes, respectively. Then the rats were sacrificed and the organs including brain, heart, liver, spleen, lung, kidneys, and testes were taken out and weighed immediately. The organ coefficients were calculated as the ratio of tissue wet weight (g) to body weight (100 g). 

#### 2.2.4. Histopathological Examination

After weighing the organs, they were immediately fixed in 10% formalin for histopathological examination. Tissue samples of M8, M5, Cr and control groups were examined to check the pathological effects caused by different treatments. The formalin fixed tissues were stored at 4 °C until examination. Tissues were processed using standard histological laboratory techniques. Using a microtome, tissue sections were cut to about a 3–4 µm thickness, then stained with hematoxylin and eosin (H&E) stain using a standard staining protocol.

#### 2.2.5. Western Blot Analysis

Brain tissue samples were frozen in liquid nitrogen and then stored at −80°C until use. Approximately 20–30 mg of tissue samples were ground with a mortar and pestle in liquid nitrogen. 100 μL of lysis buffer containing 10 mmol/L PMSF and 1 mM EDTA was then added to the tissue residue for 4 h. Then the lysate was centrifuged at 12,000 rpm for 15 min at 4 °C to obtain the supernatant. Protein concentrations of the supernatant were determined by the BCA method. The supernatant was mixed with 5× loading buffer (4:1 in volume) and boiled for five min for western blots. Polyacrylamide stacking (6%) gels and resolving (10%) gels were used to separate the proteins of different molecular weights. Immunoblots for expression of p38, JNK, p-JNK, NFκB, Nrf2, Akt, and GAPDH were detected, respectively. The Tanon 4200SF imaging processing system (Shanghai, China) was used for western blot analysis.

#### 2.2.6. Statistical Analysis

Statistical analysis was performed using SPSS 16.0 statistical software (SPSS Inc., Chicago, IL, USA). One-way ANOVAs were used for organ coefficients comparison, hematological, biochemical and hormonal analysis and probe test. ANOVAs were used to analyze the change of body weights and the MWM learning data. The Student’s *t*-test was used to compare the difference between the experimental groups and the control group. The *p* value less than or equal to 0.05 was considered as statistical significance. The results are presented as means ± standard error (SEM). 

## 3. Results

### 3.1. The Body Weight Change

**Figure 1:**
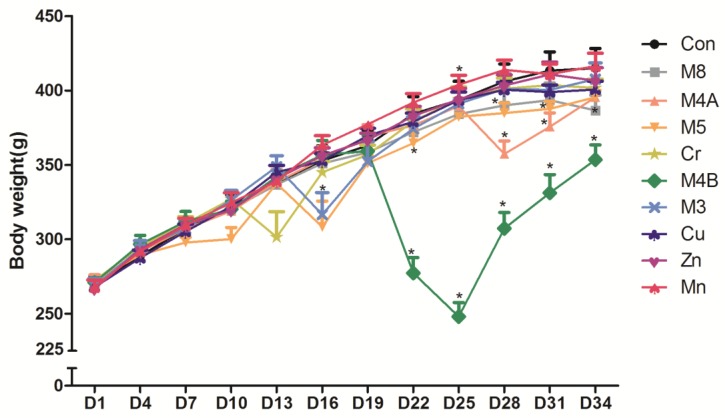
The body weight changes of rats during the experiment period. *p* < 0.05, vs*.* the control group, D1 represents the first day of the experiment, D34 represents the 34th day of the experiment, and so on. Abbreviations: Con, control group; M8: the mixture of eight common metals (Pb + Cd + Hg + Cu + Zn + Mn + Cr + Ni); M5: the mixture of five non-essential metals (Pb + Hg + Cd + Ni + Cr); M4A: the mixture of four non-essential metals (Pb + Hg + Cd + Ni); M4B: the mixture of three essential metals and one non-essential metal (Cu + Zn + Mn + Cr); M3: the mixture of three essential metals (Cu + Zn + Mn); Cr, chromium; Cu, copper; Zn, zinc; Mn, manganese.

All rats survived the duration (34 days) of the study. The rat body weight changes for each group during the entire experimental period is shown in Figure 1. The ANOVA analysis indicates that there is no difference in body weights among different groups at the beginning of the experiment (D1). But a significant time-by-group interaction was found (*p* < 0.05) later during the experiment. There was a significant difference (*p* < 0.05) in body weight as the study progressed. From the 13th day (D13) to the 34th day (D34) (except the 19th day), there was significant statistical differences in body weights among the different treatment groups. Further comparison between two groups, the body weight of the M8 group was significantly lower than that of the control group at day 34 (*p* < 0.05). On the 16th, 22nd, 28th and 31st day, the body weight of the M5 group was significantly lower than the control group (*p* < 0.01) while that of M4B and M4A groups declined from day 22 and 28 respectively compared to the control group.

### 3.2. Morris Water Maze Test

**Figure 2:**
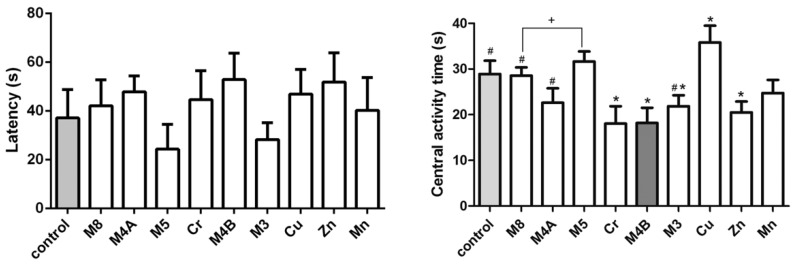
The latency of rats detected by the Morris water maze (MWM) test. * *p* < 0.05, *vs.* the control group; ^#^ *p* < 0.05 vs. the M4B group; ^+^ *p* < 0.05, M8 group vs. M5 group. Abbreviations: Con, control group; M8: the mixture of eight common metals (Pb + Cd + Hg + Cu + Zn + Mn + Cr + Ni); M5: the mixture of five non-essential metals (Pb + Hg + Cd + Ni + Cr); M4A: the mixture of four non-essential metals (Pb + Hg + Cd + Ni); M4B: the mixture of three essential metals and one non-essential metal (Cu + Zn + Mn + Cr); M3: the mixture of three essential metals (Cu + Zn + Mn); Cr, chromium; Cu, copper; Zn, zinc; Mn, manganese.

The result of the MWM test is shown in Figure 2. The M4B and the Zn group had the longest latent period compared to other treatment groups although no statistical significance was found when compared to the control group. The central activity time of the M4B group is lower than the control group, the M8, M4A and M3 groups (*p* < 0.05). 

### 3.3. Organ/Body Weight Coefficients, Hematological, Biochemical, Electrolyte and Thyroxine Parameter Analysis 

#### 3.3.1. Organ/body Weight Coefficients 

Organ/body weight coefficients of the rats are shown in Table 2. No significant differences were found in organ coefficients of the heart and liver in all treatment groups compared to the control group. In the M4B group, the coefficients of spleen, lung, kidney, brain and testis were all higher than that of the control group (*p* < 0.05). Only in the M8 and Cr groups was there an increase in lung and spleen coefficients compared to the control group (*p* < 0.05). 

#### 3.3.2. Hematological Analysis 

The hematological effect of different treatments is shown in Table 3. There is no significant difference in the WBC between the control and all other experimental groups. The WBC classification data showed that the neutrophil percentage (NE%) of M4B group was significantly higher while the lymphocyte percentage (LY%) was significantly lower than the control and all other experimental groups (*p* < 0.01). The percentage of basophil (BASO%) in the M8 and M5 groups was lower than the control group (*p* < 0.05). The Hb in the Mn group was higher than the control group (*p* < 0.05). The average erythrocyte volume (MCV) of the Cu and Zn groups was lower than that of the control group (*p* < 0.05).

#### 3.3.3. Biochemical, Electrolyte and Thyroxine Analysis 

Biochemical analysis on the liver, renal and lipid profiles is shown in Table 4. The total bilirubin (TBIL) in the M4B group was significantly higher than that in the control and the other experimental groups except for Cr group (*p* < 0.05). The total proteins (TP) in the Cu and Mn groups were significantly higher than that in the control group (*p* < 0.05). The urinary alkaline phosphatase (ALP) of the M4B group was significantly higher than the control group (*p* < 0.05). The cholesterol (CHOL) in the serum of the M4B group was significantly higher than that in the M8, M3, Cu, Zn and Mn groups. The TG in the serum of M4B group was significantly lower than that in the M4A, Zn and Mn groups. The serum high density lipoprotein (HDLC) level of the M4B group was significantly higher than that in the control and the other experimental groups. The HDLC of the M3 group was significantly lower than that in the control, M4A, M4B, Cr and Mn groups (*p* < 0.05). 

Electrolyte and thyroxine analysis is shown in Table 5. The serum calcium levels in the M4B, M5, Cu, Zn and Mn groups were significantly higher than that in the control group (*p* < 0.05). The levels of serum phosphorus in the M4B group were significantly higher than all other groups except the Mn and M5 groups (*p* < 0.05). The level of free T3 in serum of the M4B group was significantly lower than that in the M5 and Cu groups (*p* < 0.05), it is also lower than the control and all other treatment groups but without statistical significance. The level of free T4 in serum of the M4B and M4A groups was significantly higher than that in the Cu and Zn groups. The level of T3 in serum of the M5 group was significantly higher than that in the Cr, Zn and Mn groups. The serum T4 level in the M4B group was higher than that in the control and all other experimental groups but without statistical significance.

### 3.4. Histopathological Examination

**Figure 3:**
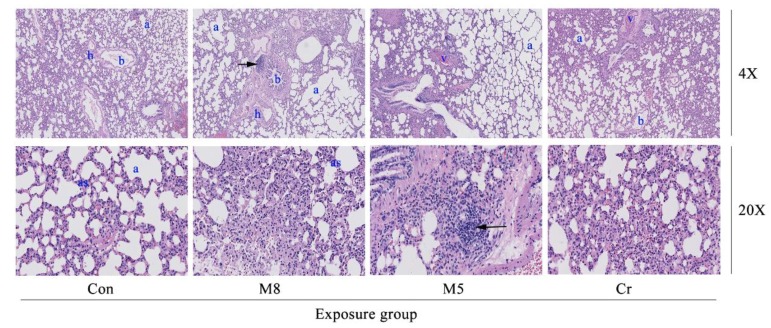
Photomicrographs of lung histopathology. The images (magnification 10×, 20×) represent the histopathological sections of the lung. Dark arrows in the M8 group (10×) represent edema and exudation around bronchi, and a large number of inflammatory cells infiltrations. Dark arrows in the M5 group (20×) represent a large number of lymphocytic infiltration around bronchi. Abbreviations: Con, control group; M8: the mixture of eight common metals (Pb + Cd + Hg + Cu + Zn + Mn + Cr + Ni); M5: the mixture of five non-essential metals (Pb+Hg+Cd+Ni+Cr); Cr, chromium; a, alveolar space; b, bronchioles; h, histiocytes; as, alveolar septae; v, veins.

The M8, M5 and Cr groups showed a mild to moderate lymphocyte aggregation in the alveolar, peribronchial, and perivascular regions (Figure 3). The M8 group showed a mild fracture and congestion on the alveolar wall. 

**Figure 4:**
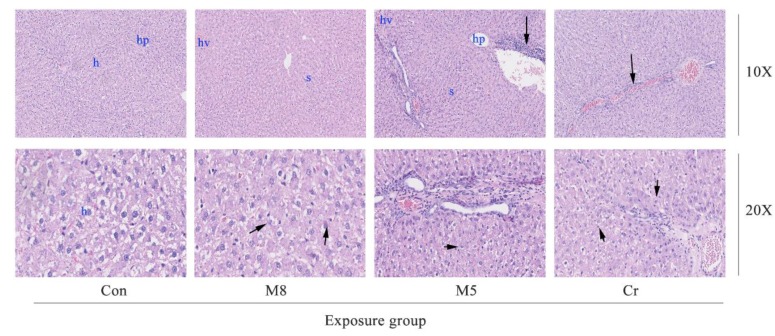
Photomicrographs of liver histopathology. The images (magnification 10×, 20×) represent the histopathological sections of the liver. Dark arrows in the M8 group (20×) represent granular degeneration; in the M5 group (10X) represent the small focal-like inflammatory cells infiltration around the portal area; in the M5 group (20X) represent lymphocyte aggregation; in the Cr group (10×) represent small focal-like inflammatory cells infiltration around the hepatic vein; in the Cr group (20×) represent hepatocyte granule degeneration and small stove liver cell shrinkage. Abbreviations: Con, control group; M8: the mixture of eight common metals (Pb + Cd + Hg + Cu + Zn + Mn + Cr + Ni); M5: the mixture of five non-essential metals (Pb + Hg + Cd + Ni + Cr); Cr, chromium; h, liver hepatocytes; hp, hepatic portal; s, sinus; hv, hepatic vein.

**Figure 5:**
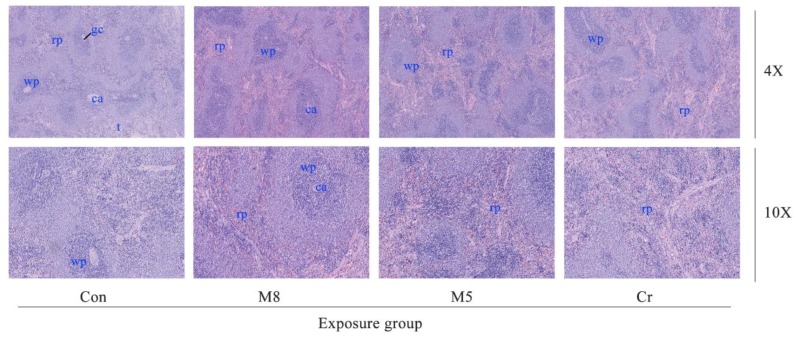
Photomicrographs of spleen histopathology. The images (magnification 4×, 10×) represent the histopathological sections of the spleen. Abbreviations: Con, control group; M8: the mixture of eight common metals (Pb + Cd + Hg + Cu + Zn + Mn + Cr + Ni); M5: the mixture of five non-essential metals (Pb + Hg + Cd + Ni + Cr); Cr, chromium; rp, red pulp; wp, white pulp; t, trabeculae; ca, central artery; gc, germinal center.

In the M8, M5 and Cr groups, the normal architecture of the liver parenchyma was distorted showing sinusoidal dilatation and congestion as well as activation of the sinusoid kupffer cell response (Figure 4). The M5 group showed lymphocytic infiltration at the periphery of the liver. The M5 and Cr groups showed small focal-like inflammatory cells infiltration. Increased red pulp area and severe congestion can be observed in the spleen of the M5 and Cr groups (Figure 5).

**Figure 6:**
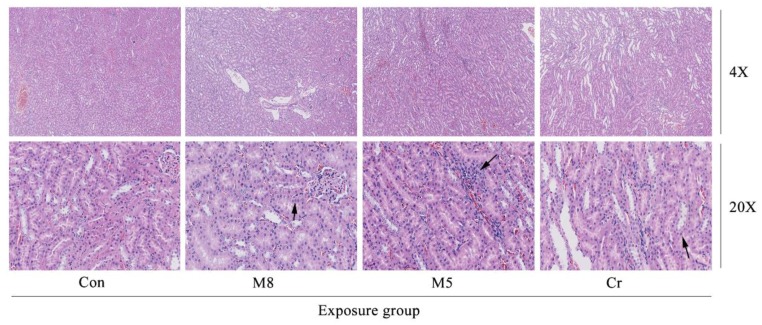
Photomicrographs of kidney histopathology. The images (magnification 4×, 20×) represent the histopathological sections of the kidney. M8 group and Cr group (20×), large areas of renal tubular epithelial cells were cloudy and swollen, significant degeneration, vacuolar degeneration of the membrane (dark arrow). M5 group (20×) a large number of inflammatory cells infiltration (dark arrow). Abbreviations: Con, control group; M8: the mixture of eight common metals (Pb + Cd + Hg + Cu + Zn + Mn + Cr + Ni); M5: the mixture of five non-essential metals (Pb + Hg + Cd + Ni + Cr); Cr, chromium.

The M8, Cr and M5 groups showed kidney inflammation (Figure 6). In the M8 group, large areas of renal tubular epithelial cells were cloudy and swollen, with vacuolar degeneration of the membrane, and focal pyknosis. Granule denaturation and membrane vacuolar degeneration were seen in the epithelial cells of the renal tubule of Cr and M5 groups. 

### 3.5. Western Blot Analysis

**Figure 7:**
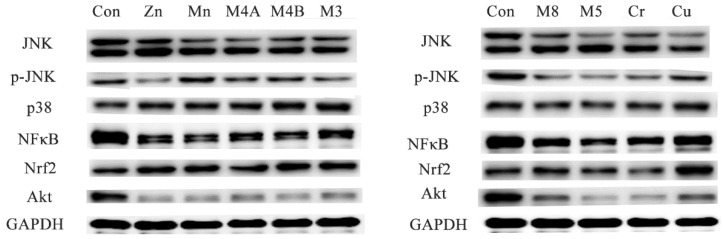
Signal protein expressions in the brain tissue. Abbreviations: Con, control group; M8: the mixture of eight common metals (Pb + Cd + Hg + Cu + Zn + Mn + Cr + Ni); M5: the mixture of five non-essential metals (Pb + Hg + Cd + Ni + Cr); M4A: the mixture of four non-essential metals (Pb + Hg + Cd + Ni); M4B: the mixture of three essential metals and one non-essential metal (Cu + Zn + Mn + Cr); M3: the mixture of three essential metals (Cu + Zn + Mn); Cr, chromium; Cu, copper; Zn, zinc; Mn, manganese.

Western blot analysis of the expressions of JNK, p-JNK, p38, NFκB, Nrf2 and Akt proteins were carried out on small sections of the brain tissue (Figure 7). There was a significant decrease in the expression of NF-κB in the brain tissue of all treatment groups when compared to the control group. The expression of p-JNK showed a significant decrease in the Zn, Cr, M3, M5 and M8 groups. Akt protein expression shows a significant decrease in all treated groups compared to the control. A significant increase of Nrf2 protein expression was observed in the brain tissue of the Zn, Mn, M4B, M3, Cu and M8 groups compared to the control group. 

## 4. Discussion

Industrialization and anthropogenic activities bring more and more heavy metal pollution [25]. Different heavy metals may simultaneously enter the human body through air, drinking water, or food. Therefore, risk assessment on the combinations of different heavy metals has more practical significance than the assessment of an individual heavy metal [22]. Recent studies indicate that fish can be used as a bio-indicator for heavy metal pollution [26]. As a major port city in China, Ningbo is known for the variety of aquatic products such as fish it offers. The health hazards of multi-heavy metal pollution through fish consumption have attracted wide attention in recent years. In previous studies, we found that the eight most common detectable heavy metals in the consumed fish in the Ningbo area are Zn, Cu, Mn, Cr, Ni, Cd, Pb and Hg, respectively [20]. The three most predominant exposed metals found in fish in the Ningbo area are: Zn, Cu, and Mn, which are all essential metals to the human body. Health effects after high dose exposure of these essential heavy metals as well as their joint effects with other non-essential heavy metals remain unclear. This is the reason why we evaluate the joint toxicities of different heavy metal combination mixtures according to the proportion of heavy metals in the consumed fish in Ningbo.

### 4.1. Systemic Toxicities

After a 34 day study, though all rats survived in all treatment groups, the systemic toxicities could be observed through body weight changes, especially in the M4B, M8, M4A, and M5 HMMs group. Animal body weight growth was significantly slower or dropped much faster at certain time points than that of the control group. Histopathological examination of the lung, liver, spleen, and kidney tissue samples of the M8, M5 and Cr groups revealed that certain damages could be observed such as: edema and exudation around the bronchi and inflammatory cells infiltration in the lungs of the M8 group; granular degeneration in the liver of the M8 group; small focal-like inflammatory cells infiltration around the portal area in the liver of the M5 group; hepatocyte granule degeneration and small stove liver cell shrinkage in the liver of the Cr group; renal tubular epithelial swelling in the kidneys of the M8 group; inflammatory cells infiltration in the kidneys of the M5 group; but no significant pathological changes could be observed in the spleen of any examined rats.

It is worthy to note that the M4B (Cu + Zn + Mn + Cr) combination was much more toxic than any other treatment, which was confirmed by more parameter abnormalities in organ/body weight coefficients, MWM test, as well as hematological, biochemical, electrolyte, thyroxine, and western-blot analysis. This phenomenon needs to be further studied because three of the four elements in the M4B mixture are essential elements for the human body, only Cr is believed to be a very toxic heavy metal. The same effects were also observed in the organ/body weight coefficients in the M4B group, the coefficients of spleen, lung, kidney, brain and testis were all higher than that of the control group, reflecting possible inflammatory edema induced in these organs.

### 4.2. Hematological, Hepatic, Renal and Spleen Toxicities

In this study, we found that the M4B mixtures increased neutrophil percentage (NE%) but decrease lymphocyte percentage (LY%), whiles the M8 and M5 mixtures resulted in a decrease in basophil percentage (BASO%). The Hb in the Mn group was higher than that in the control group. The average erythrocyte volume (MCV) of the Cu and Zn groups were lower than that of the control group. These results suggest that only a slight effect on the hematological system could be found after a 34 days experiment with the different treatments. The adverse effects of M4B, M8 and M5 mixtures on the hematological system need further confirmation.

From the results of biochemical analysis on the liver, renal and lipid profiles, the M4B mixture induced the most parameter abnormities including ALP, CHOL, HDLC increase, and TG decrease. Only in the M8 mixture was there HDLC decrease. 

Electrolyte and thyroxine analysis showed that the M4B mixture had significant effects on serum calcium, phosphorus, free T3, T4, and free T4 levels. The M5, Cu, Zn and Mn groups only affected the serum calcium levels. These results indicate the M4B mixture has a significant effect on the electrolyte and thyroxine parameters.

In previous studies, we investigated the cytotoxicity of the individual and combinations of eight heavy metals (Pb, Cd, Hg, Cu, Zn, Mn, Cr, Ni) on a human liver cell line HL7702 cells, and found that, according to the 24 h LC50 values, the toxicities ranked: Hg > Cr = Cd > Cu > Zn > Ni > Mn > Pb [22]. In this study the most toxic heavy metal in the M4B mixture is Cr; the other three heavy metals in the mixture were Cu, Zn and Mn, respectively. The M4B mixture showed a significant additive effect in toxicities compared to the M8, M5 and M4A mixtures. Both the M4B and M5 group contain the same amount of Cr. Our results suggest that Cu, Zn, Mn and Cr together generate additive or synergistic toxic effects, but Pb, Cd, Hg, Ni and Cr together may have antagonistic toxic effects. Even the M8 mixture did not generate as great a toxic effect as the M4B mixture. 

Loubières et al. [27] reported that laboratory analysis of a woman who died 12 hours after ingesting 50 mL of pure chromic acid (25 g Cr(VI)) revealed anemia symptoms. The toxicity of heavy metals is related to its ionic state. From the chemical point of view, lead acetate mixed with potassium dichromate can lead to lead chromate precipitation, and the concentration of lead ion and chromium ion dissociation by lead chromate precipitation are less than the total concentration of lead and chromium [28], presumably this is one of the reasons why the M8 and M5 mixtures generated lesser toxic effect than the M4B mixture. 

Research demonstrated that excessive exposure of chromium and manganese has an effect on thyroid functions and can cause goiter [29,30]. In this study, we found that the mixture of chromium and manganese in the M4B group can caused an increase in the serum T4 levels and free T4 levels, affecting the secretion and metabolism of thyroid hormones.

Cadmium and zinc are also known to have a variety of interactions due to the metal-binding protein metallothionein. Numerous data show that increased Zn may reduce Cd absorption and accumulation, preventing or reducing the adverse actions of Cd [31], whereas Zn deficiency can intensify Cd accumulation and toxicity. Cobbina et al. [32] suggest that the combined exposure of low concentrations of Pb + Cd + Hg, Pb + Cd or Cd + Hg had an effect on the concentration distribution of Ca, Mg, Cu and Zn in mice, and there was also a joint effect. In this study, the levels of calcium, magnesium and phosphorus in the serum of the M5 group were slightly affected while those of serum calcium and phosphorus in the M4B group were significantly affected.

In this study, using the animal study protocols approved by the Ningbo University Institutional Animal Care and Use Committee, we checked the histopathological effect in the M8, M5, Cr and control groups. Histopathological examination of the lung tissue showed wide spread pathology in the lungs of the M8, M5, and Cr groups. The most prominent feature in all dose groups was the inflammatory cells infiltration consisting of lymphocytes, neutrophils and macrophages. The aggregates of these infiltrates are interstitial and predominantly in a peri-bronchial location but they extend to the alveolar wall. Severe congestion of the spleen of the Cr and M5 groups suggested that the heavy metal mixtures lead to spleen damage.

### 4.3. Neurotoxicity 

The MWM results showed that the incubation period of the Zn, M4B and Cr groups were much longer than that of the control group though there was no significant difference. Water maze latency period represents the animal space learning and memory ability good or not, a short incubation period indicates that the learning and memory ability of the animal is good, but the water maze latency period may also affect by the animal's swimming speed. Therefore, the latency period can’t be used as a single indicator to judge the memory ability. Animals swim roundabout the pool edge, and then a longer center activity time may also reflect a better space memory [33]. The central activity time of the M4B, Cr, Zn, and M3 groups were all lower than the control group, suggesting that heavy metals may lead to reduced spatial memory. Previous studies have shown that Cr seems to exert its genetic effects by binding directly to DNA. A clinical study found strong DNA oxidative damage from the urinary samples of a patient who ingested two to three grams of potassium dichromate in a suicide attempt [34]. Exposure to high Mn levels is known to cause adverse neurological effects in humans [35]. Combined with our results, both individual heavy metals (including essential and non-essential elements) and combined HMMs have the potential to induce neurological damages.

NF-κB (nuclear factor kappa-light-chain-enhancer of activated B cells) is a protein complex that controls transcription of DNA, cytokine production and cell survival [36]. Current studies demonstrated that NF-κB is important for learning and memory in multiple organisms including fruit flies [37] and mice [38,39]. Protein kinase B (PKB), known as Akt, plays an important role in multiple cellular processes such as apoptosis, cell proliferation, transcription and cell migration. Research showed that Akt is also required for recognition memory and spatial learning [40]. C-Jun N-terminal kinases (JNKs) are considered to be involved in the diversified biological and pathological functions during brain development and pathogenesis of diseases [41]. JNK also plays an important role in the regulation of microtubule stability in neurons [42]. NF-E2-related factor (Nrf2), a transcription factor, has been shown to play an essential role in the antioxidant response element (ARE)-mediated expression of phase 2 detoxifying enzymes and stress-inducible genes [43]. Research evidence shows that Nrf2 gene transfer reduces learning and memory impairment in APP/PS1 mice [44]. In this study, western-blot analysis revealed a significant decrease in the expression of NFκB and Akt in brain tissue of all treatment groups compared to the control group. The expression of p-JNK is also significantly decreased in the Zn, Cr, M3, M5 and M8 groups. Nrf2 protein expressions in brain tissue increase significantly in the Zn, Mn, M4B, M3, Cu and M8 groups compared to the control group. Why NFκB, Akt and p-JNK are down-regulated whereas Nrf2 is up-regulated? Is there any relationship between these signal protein expressions and the neurological damages induced by the heavy metals? All these questions need to be further studied in order to elucidate the detail mechanisms.

In summary, based on the proportion of heavy metals consumed in aquatic products in Ningbo area, toxicities after a short-term oral repeated-administration of different individual heavy metals or their mixtures in rats were evaluated. No animal deaths were found in all treatment groups during the 34 days experiment period. The M4B mixture showed the most toxic joint effects on animals and was even more toxic than that of the M8 mixture. Toxic effects that were observed include abnormalities in hematological, hepatic, renal functions, lipid metabolism, serum electrolyte and thyroid hormone levels. Certain adverse effects on the learning and memory ability of rats were also observed in the Zn, M4B and Cr groups. Overall, our study showed that the M4B group had higher cytotoxic and genotoxic effects than the M8 group in vivo studies under the same treatment dose. SD rats are often used to study the adverse effects of environmental pollutants such as heavy metals, which is useful for providing theoretical basis and guidance for the early prevention and control on heavy metal induced toxicities [45,46]. Due to the uncertainty of extrapolation of experimental data from animals to human body [47], further more studies using histochemical and molecular biological methods will be needed for elaborating the detail mechanisms of these findings.

## 5. Concussions

In this study, we found that combination of different HMMs showed certain adverse effects on the hematologic, hepatic, renal and neurobehavioral function, and could also disturb electrolyte and lipid balance in rats. This suggests that joint toxicity or interaction patterns among different heavy metals should be taken into consideration during the risk assessment for the exposure to multi-heavy metal simultaneously. The detail mechanisms of M4B combination generated much higher toxic effects than any other combination mixtures or individual heavy metal needs to be further evaluated. To mimic human exposure, further studies are also necessary to evaluate the combined effects after a long-term low-level HMMs exposure in animals. 

## Figures and Tables

**Table 1:** Composition of compound dose for each group.

Group	Compound Dose (mg/Kg Body Weight)							
	(CH_3_COO)_2_Pb·3H_2_O	CdCl_2_·2.5H_2_O	CH_3_HgCl	NiCl_2_·6H_2_O	K_2_Cr_2_O_7_	CuSO_4_·5H_2_O	ZnSO_4_·7H_2_O	MnCl_2_·4H_2_O
Con	0	0	0	0	0	0	0	0
M8	0.087	0.193	0.009	0.874	1.681	8.831	33.840	4.484
M5	0.087	0.193	0.009	0.874	1.681	0	0	0
M4A	0.087	0.193	0.009	0.874	0	0	0	0
M4B	0	0	0	0	1.681	8.831	33.840	4.484
M3	0	0	0	0	0	8.831	33.840	4.484
Cr	0	0	0	0	1.681	0	0	0
Cu	0	0	0	0	0	8.831	0	0
Zn	0	0	0	0	0	0	33.840	0
Mn	0	0	0	0	0	0	0	4.484

Abbreviations: Con, control; M8: the mixture of eight common metals (Pb + Cd + Hg + Cu + Zn + Mn + Cr + Ni); M5: the mixture of five non-essential metals (Pb + Hg + Cd + Ni + Cr); M4A: the mixture of four non-essential metals (Pb + Hg + Cd + Ni); M4B: the mixture of three essential metals and one non-essential metal (Cu + Zn + Mn + Cr); M3: the mixture of three essential metals (Cu + Zn + Mn); Cr, chromium; Cu, copper; Zn, zinc; Mn, manganese.

**Table 2:** Organ/body weight coefficients of rats after treatment with different heavy metal compositions.

Group	Organ/Body Weight Coefficients (% Weight)						
	Heart	Liver	Spleen	Lung	Kidney	Brain	Testis
Con	0.38 ± 0.01	3.56 ± 0.08	0.18 ± 0.01	0.41 ± 0.01	0.75 ± 0.01	0.48 ± 0.02	0.70 ± 0.02
M8	0.39 ± 0.03	3.49 ± 0.05	0.19 ± 0.01	0.46 ± 0.01 *	0.77 ± 0.04	0.50 ± 0.02	0.75 ± 0.05
M4A	0.40 ± 0.02	3.46 ± 0.09	0.19 ± 0.01	0.44 ± 0.02	0.74 ± 0.03	0.49 ± 0.02	0.73 ± 0.05
M5	0.37 ± 0.01	3.64 ± 0.05	0.19 ± 0.01	0.44 ± 0.01	0.79 ± 0.01	0.50 ± 0.01	0.76 ± 0.03
Cr	0.41 ± 0.02	3.61 ± 0.09	0.21 ± 0.01 *	0.45 ± 0.01	0.76 ± 0.02	0.48 ± 0.01	0.78 ± 0.03
M4B	0.41 ± 0.02	3.62 ± 0.09	0.22 ± 0.01 *	0.52 ± 0.02 *	0.82 ± 0.01 *	0.56 ± 0.02 *	0.87 ± 0.03 *
M3	0.38 ± 0.01	3.51 ± 0.06	0.19 ± 0.01	0.47 ± 0.01 *	0.75 ± 0.02	0.46 ± 0.02	0.74 ± 0.02
Cu	0.38 ± 0.02	3.54 ± 0.07	0.19 ± 0.01	0.44 ± 0.01	0.75 ± 0.02	0.48 ± 0.01	0.77 ± 0.02
Zn	0.41 ± 0.02	3.68 ± 0.06	0.18 ± 0.01	0.43 ± 0.02	0.78 ± 0.02	0.47 ± 0.01	0.72 ± 0.03
Mn	0.39 ± 0.02	3.49 ± 0.06	0.18 ± 0.01	0.43 ± 0.01	0.75 ± 0.03	0.47 ± 0.01	0.71 ± 0.01

Notes: Results given as mean ± SD (* *p* < 0.05 vs. control). Abbreviations: Con, control; M8: the mixture of eight common metals (Pb + Cd + Hg + Cu + Zn + Mn + Cr + Ni); M5: the mixture of five non-essential metals (Pb + Hg + Cd + Ni + Cr); M4A: the mixture of four non-essential metals (Pb + Hg + Cd + Ni); M4B: the mixture of three essential metals and one non-essential metal (Cu + Zn + Mn + Cr); M3: the mixture of three essential metals (Cu + Zn + Mn); Cr, chromium; Cu, copper; Zn zinc; Mn, manganese.

**Table 3:** Hematological effects of different treatments.

Group	WBC	NE%	LY%	BASO%	RBC	Hb	MCV	MCHC	PLT	MPV
Con	8.30 ± 0.71	17.03 ± 1.96	76.43 ± 2.18	0.10 ± 0.03	7.53± 0.10	142.50± 1.03	60.97± 0.54	311.00± 1.21	759.50± 111.26	7.07± 0.16
M8	7.53 ± 0.60	17.82 ± 2.04	76.23 ± 2.47	0.02 ± 0.02 *	7.79 ± 0.24	145.17 ± 3.01	60.80 ± 1.05	307.33 ± 2.06	728.83 ± 120.80	7.23 ± 0.10
M5	9.10 ± 1.38	13.65 ± 0.62	81.65 ± 0.82	0.02 ± 0.02 *	7.89 ± 0.10	146.00 ± 1.10	59.33 ± 0.55	312.00 ± 1.57	891.00 ± 36.62	6.93 ± 0.14
Cr	8.68 ± 0.81	17.12 ± 4.63	76.48 ± 6.13	0.10 ± 0.03	7.57 ± 0.12	141.17 ± 1.58	61.17 ± 0.71	305.67 ± 1.09 *	853.67 ± 76.48	6.83 ± 0.13
Cu	8.85 ± 0.64	17.25 ± 2.45	76.00 ± 2.83	0.07 ± 0.03	8.08 ± 0.18 *	148.50 ± 2.53	58.88 ± 0.55 *	312.17 ± 1.85	903.33 ± 13.39 *	6.77 ± 0.19
Zn	9.18 ± 0.89	14.52 ± 1.84	78.78 ± 2.10	0.08 ± 0.02	8.04 ± 0.16 *	148.17 ± 2.27	59.35 ± 0.37 *	311.00 ± 2.22	860.83 ± 41.34	6.80 ± 0.21
Mn	9.52 ± 0.98	14.17 ± 1.42	80.13 ± 1.90	0.10 ± 0.00	7.91 ± 0.14	149.17 ± 2.63 *	60.65 ±0.92	310.83 ± 1.38	901.17 ± 43.06	6.80 ± 0.22
M4A	8.68 ± 0.96	17.27 ± 2.59	76.28 ± 2.46	0.07 ± 0.02	7.64 ± 0.13	143.67 ± 1.71	60.30 ± 0.58	311.50 ± 1.41	848.33 ± 53.73	6.82 ± 0.19
M4B	8.43 ± 1.24	31.84 ± 3.57 *	44.60 ± 11.74 *	0.08 ± 0.02	7.58 ± 0.08	142.67 ± 1.23	60.73 ± 0.44	309.67 ± 1.43	903.83 ± 53.06 *	6.50 ± 0.18
M3	8.17 ± 0.35	15.57 ± 1.70	77.63 ± 1.36	0.08 ± 0.02	7.81 ± 0.19	144.50 ± 3.38	59.07 ± 0.28	312.67 ± 2.22	723.83 ± 100.47	6.55 ± 0.21

Results given as mean ± SD (* *p* < 0.05 vs. control). Abbreviations: Con, control; WBC, white blood cells (×10^9^/L); NE%, neutrophil percentage; LY%, lymphocyte percentage; BASO%, percentage of granulocytes; RBC, red blood cell (×10^12^/L); Hb, hemoglobin (g/L); MCV, average erythrocyte volume (fl); MCHC, mean corpuscular hemoglobin concentration (g/L); PLT, platelet count (×10^9^/L); MPV, mean platelet volume (fl); M8: the mixture of eight common metals (Pb + Cd + Hg + Cu+ Zn + Mn + Cr + Ni); M5: the mixture of five non-essential metals (Pb + Hg + Cd + Ni + Cr); M4A: the mixture of four non-essential metals (Pb + Hg + Cd + Ni); M4B: the mixture of three essential metals and one non-essential metal (Cu + Zn + Mn + Cr); M3: the mixture of three essential metals (Cu + Zn + Mn) ;Cr, chromium; Cu, copper; Zn, zinc; Mn, manganese.

**Table 4:** The biochemical analysis of the liver, renal and lipid profiles.

Group	Liver profile								Renal profile		Lipid profile				
	TBIL	TP	ALB	GLOB	ALT	AST	ALP	UR/Cr		URCA		CHOL	TG	HDLC	LDLC
Con	0.27 + 0.20	51.38 + 2.70	34.58 + 0.30	16.80 + 2.80	70.00 + 3.31	110.67 + 7.23	173.50 + 11.51	0.26 + 0.02		140.17 + 15.53		1.27 + 0.10	0.93 + 0.13	0.67 + 0.05	0.23 + 0.02
M8	0.10 + 0.31	52.94 + 0.58	34.00 + 0.47	18.94 + 0.15	75.20 + 5.42	159.00 + 38.07	188.00 + 26.98	0.29 + 0.03		120.20 + 11.81		1.08 + 0.06	0.86 + 0.19	0.60 + 0.04	0.21 + 0.01
M5	0.15 + 0.14	55.68 + 0.49	35.57 + 0.51	20.12 + 0.22	90.33 + 17.63	222.50 + 69.25	226.50 + 21.54	0.30 + 0.01		133.17 + 17.55		1.23 + 0.05	1.02 + 0.11	0.64 + 0.02	0.22 + 0.01
Cr	0.65 + 0.27	54.17 + 0.85	33.85 + 0.47	20.32 + 0.44	67.00 + 4.87	107.67 + 14.06	198.17 + 20.24	0.28 + 0.02		95.33 + 9.42 *		1.30 + 0.07	0.87 + 0.11	0.66 + 0.04	0.22 + 0.01
Cu	0.20 + 0.28	57.68 + 0.60 *	35.68 + 0.36	22.00 + 0.37	71.00 + 1.61	118.50 + 11.04	232.00 + 23.68	0.30 + 0.01		88.17 + 6.14 *		1.20 + 0.04	0.92 + 0.09	0.63 + 0.02	0.23 + 0.01
Zn	0.05 + 0.23	56.65 + 1.16	35.10 + 0.51	21.55 + 0.77	66.50 + 3.09	119.17 + 16.25	175.50 + 12.24	0.30 + 0.02		80.83 + 13.37 *		1.10 + 0.06	1.11 + 0.17	0.59 + 0.03	0.21 + 0.02
Mn	0.30 + 0.28	57.32 + 1.53 *	35.57 + 0.91	21.75 + 0.69	69.67 + 2.35	115.83 + 13.47	195.00 + 21.41	0.28 + 0.02		122.00 + 16.29		1.14 + 0.19	1.03 + 0.08	0.65 + 0.03	0.19 + 0.04
M4A	0.32 + 0.20	53.63 + 1.45	34.00 + 0.50	19.63 + 1.31	71.67 + 3.87	140.67 + 25.29	158.00 + 11.17	0.27 + 0.02		102.50 + 14.57		1.26 + 0.06	0.96 + 0.13	0.64 + 0.04	0.22 + 0.01
M4B	1.06 + 0.37 *	54.70 + 1.36	35.04 + 0.51	19.66 + 1.23	79.80 + 2.94	113.20 + 19.07	242.00 + 18.17 *	0.31 + 0.02 *		121.60 + 15.62		1.49 + 0.053	0.62 + 0.08	0.81 + 0.03 *	0.26 + 0.01 *
M3	0.27 + 0.25	53.55 + 1.12	34.37 + 0.30	19.18 + 1.09	63.33 + 3.88	143.00 + 32.73	152.67 + 15.17	0.28 + 0.02		114.67 + 18.99		1.07 + 0.04	0.94 + 0.04	0.55 + 0.02	0.22 + 0.01

Results given as mean ± SD (* *p* < 0.05 versus control). Abbreviations: TBIL, total bilirubin (μmol/L); TP, total protein (g/L); ALB, albumin (g/L); GLOB, globulin (g/L); ALT, alanine aminotransferase (U/L); AST, aspartate transaminase (U/L); ALP, alkaline phosphatase (U/L); UR/Cr , the ratio of urea to nitrogen and creatinine; URCA, uric acid (μmmol/L); CHOL, total cholesterol (mmol/L); TG, triglycerides (mmol/L); HDLC, high-density lipoprotein (mmol/L); LDLC, low density lipoprotein (mmol/L); Con, control group; M8: the mixture of eight common metals (Pb + Cd + Hg + Cu + Zn + Mn + Cr + Ni); M5: the mixture of five non-essential metals (Pb + Hg + Cd + Ni + Cr); M4A: the mixture of four non-essential metals (Pb + Hg + Cd + Ni); M4B: the mixture of three essential metals and one non-essential metal (Cu + Zn + Mn + Cr); M3: the mixture of three essential metals (Cu + Zn + Mn) ;Cr, chromium; Cu, copper; Zn, zinc; Mn, manganese.

**Table 5:** Serum electrolyte and thyroxine parameter analysis.

Group	Electrolyte Parameters					Thyroxine Parameters			
	Na	Ca	Mg	PHOS	Fe	FT3	FT4	T3	T4
Con	139.47 ± 0.61	2.64 ± 0.02	1.12 ± 0.04	2.59 ± 0.08	36.17 ± 1.14	2.07 ± 0.01	12.31 ± 0.48	0.61 ± 0.03	56.73 ± 2.05
M8	139.16 ± 0.14	2.72 ± 0.03	1.16 ± 0.02	2.63 ± 0.12	35.80 ± 0.97	2.04 ± 0.15	12.41 ± 0.92	0.65 ± 0.04	57.02 ± 3.87
M5	140.05 ± 0.36	2.79 ± 0.04	1.21 ± 0.04	2.77 ± 0.10	34.83 ± 1.33	2.10 ± 0.10	10.75 ± 0.56	0.69 ± 0.03	48.71 ± 0.10
Cr	140.37 ± 0.38	2.71 ± 0.04	1.08 ± 0.03	2.70 ± 0.08	36.50 ± 3.42	1.94 ± 0.06	12.03 ± 0.70	0.58 ± 0.02	54.76 ± 3.32
Cu	141.07 ± 0.22	2.78 ± 0.03	1.12 ± 0.02	2.68 ± 0.05	32.67 ± 1.36	2.10 ± 0.07	11.57 ± 0.73	0.63 ± 0.04	55.04 ± 3.37
Zn	140.73 ± 0.47	2.80 ± 0.05	1.17 ± 0.04	2.63 ± 0.09	36.83 ± 2.18	1.89 ± 0.07	10.61 ± 0.35	0.60 ± 0.02	58.592 ± 4.56
Mn	124.70 ± 15.01	2.74 ± 0.04	1.19 ± 0.05	2.78 ± 0.05	35.83 ± 1.45	1.85 ± 0.07	11.93 ± 0.70	0.55 ± 0.02	56.43 ± 5.29
M4A	140.10 ± 0.59	2.69 ± 0.05	1.12 ± 0.03	2.67 ± 0.021	36.17 ± 1.40	1.97 ± 0.18	13.04 ± 1.03	0.62 ± 0.03	56.42 ± 3.28
M4B	141.32 ± 0.61	2.76 ± 0.02 *	1.21 ± 0.06	2.73 ± 0.08 *	45.00 ± 3.44	1.80 ± 0.08	13.64 ± 0.95	0.62 ± 0.04	70.67 ± 6.26 *
M3	139.60 ± 0.90	2.73 ± 0.03	1.15 ± 0.03	2.68 ± 0.04	37.83 ± 1.42	1.99 ± 0.03	11.90 ± 1.05	0.63 ± 0.03	51.78 ± 4.41

Results given as mean ± SD (* *p* < 0.05 vs control). Abbreviations: Na (mmol/L), Ca (mmol/L), Mg (mmol/L), PHOS (mmol/L), Fe (μmmol/L), T3, triiodothyronine (μg/L); T4, thyroid hormone (μg/L); FT3, free T3 (ng/L); FT4, free T4 (ng/L); Con, control group; M8: the mixture of eight common metals (Pb + Cd + Hg + Cu + Zn + Mn + Cr + Ni); M5: the mixture of five non-essential metals (Pb + Hg + Cd + Ni + Cr); M4A: the mixture of four non-essential metals (Pb + Hg + Cd + Ni); M4B: the mixture of three essential metals and one non-essential metal (Cu + Zn + Mn + Cr); M3: the mixture of three essential metals (Cu + Zn + Mn); Cr, chromium; Cu, copper; Zn, zinc; Mn, manganese.
